# Supplementary material for: Statistical detection of selfish mining in proof-of-work blockchain systems
Source: Sci Rep. 2024 Mar 15;14:6251. doi: 10.1038/s41598-024-55348-3 (PMC10943236; doi:10.1038/s41598-024-55348-3)
Supplement: Supplementary file 1 — Supplementary Information. [file 41598_2024_55348_MOESM1_ESM.pdf]

Supplementary Information for Statistical Detection of Selfish Mining in Proof-of-Work Blockchain Systems

Sheng-Nan Li, Carlo Campajola, Claudio J. Tessone

1 Robustness Check

As mentioned in the main text, block diffusion times due to network delay are a potential alternative explanation for the anomalies we detect in miners' rates of successive block discoveries. In a nutshell, the network delay could give some advantage to the previous block miner to start mining the next block a bit earlier than others. To verify that, compared with the larger average interval of block creation, the influence of network delay on miners' probability of block discovery is negligible in the real-world blockchain system, we further evaluate the effect of network delay on an honest miner's advantage of mining consecutive blocks. According to the PoW protocol, the miner's discovery of each block should be independent and continuously occur at a fixed rate λ. It determines that block creation in PoW is a Poisson process and the time between the creation of a new block follows the exponential distribution. For example, in Bitcoin, the network adjusts the difficulty to maintain the average inter-block time equal to 10 mins, which can be denoted as $\tau_b = \lambda^{-1} = 600$ seconds. In addition, the probability of mining a new block is proportional to the miner's share of the network's computational power, h. Thus, we adjust the rate parameter to $h_i\lambda$ as the block mining rate for the miner i with the hashing power equal to h_i. Thus, without doing any selfish mining behaviour, the probability that the previous block miner i continuously mines the next block before its block diffuses in the network could be presented as,

$$\alpha_i = 1 - e^{-h_i \lambda \tau_{nd}} \tag{1}$$

where τ_{nd} is the average block diffusion time (also named as network delay) in the system. The value of α reflects the advantage of a block miner with certain hashing power h_i to continuously discover blocks only on account of the network delay.

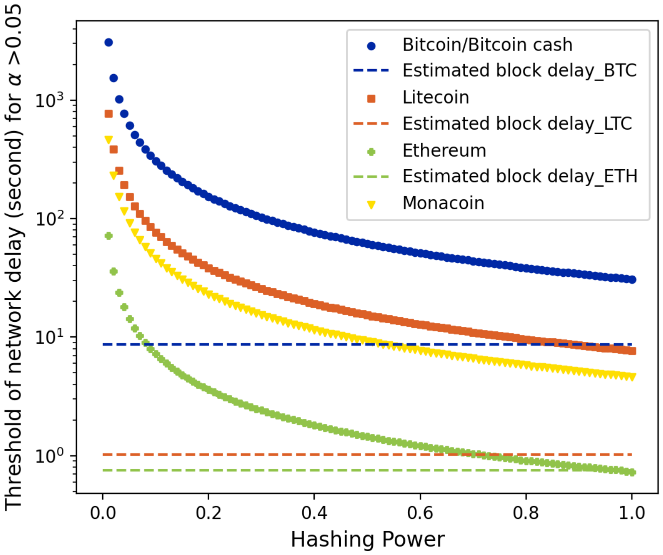

Supplementary Figure S1: Threshold of network delay needed by honest miners with different hashing power to have a certain advantage of mining consecutive blocks in different real-world networks.

As shown in Supplementary Figure S1, in the Bitcoin and Litecoin systems, miners could never have even 5% advantage under the current network delay. Even in systems with lower block intervals, like Ethereum where the network delay is estimated as 0.75 seconds, the mining entities need more than 90% of the hashing power to get the advantage. Data of the estimated block delay are taken from [2, 1]. Even though we do not know the block delay in Bitcoin cash and Monacoin, one can easily find that under any reasonable network delay miners rarely gain a significant advantage from it.

To further check the robustness of our detection under real-world network conditions, we introduce a null model to generate synthetic sequences of miner identities using an autocorrelated process induced only by network delay. Calling $X(t) \in \{1, \ldots, i\}$ the identifier of the miner that mines block t, we simulate the sequence,

$$X(t+1)_{|X(t)=i} = \eta_t i + (1 - \eta_t)\zeta(t)$$

where

- $X(t+1)_{|X(t)=i} \in \{1, \ldots, i\}$ is the next miner of the process X conditional on $X(t) = i$
- $\eta_t = 1$ with probability α_i and 0 otherwise
- $\zeta(t) = j$ with probability h_j, where h_j is miner j's fraction of the total hashrate
- $X(1) = \zeta(0)$

Basically, at each timestep there are 2 options: either copy from the last miner (if $\eta_t = 1$) or sample a new miner (if $\eta_t = 0$). The new miner is sampled according to the hashrates we estimate.

The α_i parameter (as Equ. 1), giving the probability that the previous miner i could also be the miner of the next block only because of the network delay, determines the autocorrelation in the time series. If the network has a higher latency, the α_i is larger.

For each time period analysed in the main text, we then compute the 5% critical level for the number of abnormal miners that would result from applying our testing procedure in a context where abnormal sequences may just be the result of network delay induced autocorrelations.

We generate 100 simulated time series of miner identities in each system by setting the corresponding parameters of network delay and block interval, and we compare our detection result with the detection applied to the simulated data. As shown by the grey area in Fig. 7 (in the main body of the paper), which shows the 5% significance critical level under the network delay null obtained by bootstrap, in each subperiod, only few abnormal miners should be expected, even in systems with low block intervals such as Ethereum and Monacoin. Therefore, our detection method is robust under most real-world system conditions.

References

[1] E. Fadda, J. He, C. J. Tessone, and P. Barucca. Consensus formation on heterogeneous networks. *EPJ Data Science*, 11(1):34, 2022.

[2] A. Gervais. *On the security, performance and privacy of proof of work blockchains.* PhD thesis, ETH Zurich, 2016.
